# Supplementary material for: Characteristics, clinical outcomes, and mortality of older adults living with HIV receiving antiretroviral treatment in the sub-urban and rural areas of northern Thailand
Source: PLoS One. 2023 Mar 23;18(3):e0271152. doi: 10.1371/journal.pone.0271152 (PMC10035908; doi:10.1371/journal.pone.0271152)
Supplement: Attachment — Submitted filename: Plos One review.docx [file pone.0271152.s002.docx]

This manuscript presents valuable information namely that older patients with HIV infection, despite a very high rate of virological suppression, have increased mortality over time if the CD4 cell count is <200. However, those with CD4 of 200-499 seem to have mortality similar to those with a CD4 ≥500.

It also provides important data about the successful deployment of ART in older patients in Thailand with a very high rate of virologic suppression.

Introduction

The introduction is too long

Relating to this sentence: “As a result of the physiologic change from aging, we hypothesize that the HIV treatment outcome and mortality of OALHIV might be different from PLHIV at a younger age.” Was the hypothesize that older patients were merely different? In what way? Were worse ART treatment outcomes expected or better? Was the expectation about overall survival?

Methods

What role does survivorship bias possibly play here? Please discuss and acknowledge as a limitation.

Results

Do we know what proportion of patients agreed to participate versus the number approached for entry? If there was a significant rate of refusal, what does this mean in terms of whether this cohort is representative of the larger patient population.

The authors should describe how they dealt with missing viral load tests. Were patients with missing viral load data excluded from the virologic suppression analysis or was there an assumption made about their virologic outcome (for example, assumed to be suppressed)?

How do you reconcile the high rate of virologic suppression reported with the higher rate of mortality in patients with CD4<200? Were these mainly non-AIDS related deaths?

Discussion

I think the discussion should be rewritten for clarity and it should be shorter.

I don’t think you present evidence to support this sentence: “The excess mortality and morbidity could be preventable and/or manageable if detected and intervened in a timely manner.”

Figures:

I would not use more than one decimal place as this level of precision is not useful (for example, 99.59% viral suppression).
